# Supplementary material for: OTX Genes in Adult Tissues
Source: Int J Mol Sci. 2023 Nov 30;24(23):16962. doi: 10.3390/ijms242316962 (PMC10707059; doi:10.3390/ijms242316962)
Supplement: Supplementary file 1 [file ijms-24-16962-s001.zip › Supplementary Figure 1 - OTX1 Result Summary BioGRID.pdf]

BioGRID COVID-19 Coronavirus Curation Project (https://thebiogrid.org/project/3)
Search BioGRID for SARS-CoV-2 Protein Interactions (https://thebiogrid.org/search.php?search=SARS-CoV-2*&organism=2697049) | Download SARS-CoV-2 and Coronavirus-Related Interactions (https://downloads.thebiogrid.org/File/BioGRID/Latest-Release/BIOGRID-PROJECT-covid19_coronavirus_project-LATEST.zip)

OTX1

Homo sapiens

orthodenticle homeobox 1

- CRISPR Database (https://orcs.thebiogrid.org/Gene/5013)
- VEGA (http://vega.sanger.ac.uk/id/OTTHUMG00000129454)
- OMIM (http://www.ncbi.nlm.nih.gov/omim/600036)
- HGNC (https://www.genenames.org/data/gene-symbol-report/#!/hgnc_id/HGNC:8521)
- Alliance of Genome Resources (https://www.alliancegenome.org/gene/HGNC:8521)
- Entrez Gene (http://www.ncbi.nlm.nih.gov/gene/5013)
- RefSeq (http://www.ncbi.nlm.nih.gov/sites/entrez?db=protein&cmd=DetailsSearch&term=NP_001186699+OR+NP_055377+OR+XP_006712088)
- UniprotKB (http://www.uniprot.org/uniprot/P32242)
- Ensembl (http://www.ensembl.org/Gene/Summary?g=ENSG00000115507)
- HPRD (http://www.hprd.org/protein/08964)

Download Curated Data for this Protein

Switch View: Interactors 149 Interactions 186 Network

Showing **1** to **149** of **149** unique interactors

Filter Interactions... ADV

Interactor	Evidence
MDFI (https://thebiogrid.org/110353/summary/homo-sapiens/mdfi.html)	4 View
GRN (https://thebiogrid.org/109153/summary/homo-sapiens/grn.html)	3 View

Interactor	Evidence
KRT81 (https://thebiogrid.org/110085/summary/homo-sapiens/krt81.html)	3 View
KRTAP4-12 (https://thebiogrid.org/123756/summary/homo-sapiens/krtap4-12.html)	3 View
RBPMS (https://thebiogrid.org/116220/summary/homo-sapiens/rbpms.html)	3 View
RGS20 (https://thebiogrid.org/114161/summary/homo-sapiens/rgs20.html)	3 View
ADAMTSL4 (https://thebiogrid.org/120002/summary/homo-sapiens/adamtsl4.html)	2 View
CHIC2 (https://thebiogrid.org/117716/summary/homo-sapiens/chic2.html)	2 View
COMP (https://thebiogrid.org/107706/summary/homo-sapiens/comp.html)	2 View
KRTAP1-3 (https://thebiogrid.org/123601/summary/homo-sapiens/krtap1-3.html)	2 View
KRTAP1-5 (https://thebiogrid.org/123806/summary/homo-sapiens/krtap1-5.html)	2 View
KRTAP10-1 (https://thebiogrid.org/132128/summary/homo-sapiens/krtap10-1.html)	2 View
KRTAP10-11 (https://thebiogrid.org/132129/summary/homo-sapiens/krtap10-11.html)	2 View
KRTAP10-5 (https://thebiogrid.org/132131/summary/homo-sapiens/krtap10-5.html)	2 View
KRTAP10-8 (https://thebiogrid.org/132132/summary/homo-sapiens/krtap10-8.html)	2 View
KRTAP10-9 (https://thebiogrid.org/132127/summary/homo-sapiens/krtap10-9.html)	2 View
KRTAP12-2 (https://thebiogrid.org/131684/summary/homo-sapiens/krtap12-2.html)	2 View
KRTAP12-4 (https://thebiogrid.org/132135/summary/homo-sapiens/krtap12-4.html)	2 View
KRTAP3-2 (https://thebiogrid.org/123808/summary/homo-sapiens/krtap3-2.html)	2 View
KRTAP3-3 (https://thebiogrid.org/124457/summary/homo-sapiens/krtap3-3.html)	2 View

Interactor	Evidence
KRTAP4-11 (https://thebiogrid.org/575632/summary/homo-sapiens/krtap4-11.html)	2 View
KRTAP5-6 (https://thebiogrid.org/136221/summary/homo-sapiens/krtap5-6.html)	2 View
KRTAP5-9 (https://thebiogrid.org/110044/summary/homo-sapiens/krtap5-9.html)	2 View
KRTAP9-2 (https://thebiogrid.org/123810/summary/homo-sapiens/krtap9-2.html)	2 View
LCE1B (https://thebiogrid.org/131639/summary/homo-sapiens/lce1b.html)	2 View
RGS17 (https://thebiogrid.org/117744/summary/homo-sapiens/rgs17.html)	2 View
SPRY2 (https://thebiogrid.org/115547/summary/homo-sapiens/spry2.html)	2 View
TENC1 (https://thebiogrid.org/116951/summary/homo-sapiens/tenc1.html)	2 View
VWC2L (https://thebiogrid.org/135334/summary/homo-sapiens/vwc2l.html)	2 View
ADAM12 (https://thebiogrid.org/113731/summary/homo-sapiens/adam12.html)	1 View
ALPP (https://thebiogrid.org/106751/summary/homo-sapiens/alpp.html)	1 View
ANKRD10 (https://thebiogrid.org/120748/summary/homo-sapiens/ankrd10.html)	1 View
ARHGEF17 (https://thebiogrid.org/115166/summary/homo-sapiens/arhgef17.html)	1 View
ARID5A (https://thebiogrid.org/116074/summary/homo-sapiens/arid5a.html)	1 View
ATG9B (https://thebiogrid.org/130263/summary/homo-sapiens/atg9b.html)	1 View
BHLHB9 (https://thebiogrid.org/123320/summary/homo-sapiens/bhlhb9.html)	1 View
C10ORF55 (https://thebiogrid.org/136015/summary/homo-sapiens/c10orf55.html)	1 View
C11ORF1 (https://thebiogrid.org/122286/summary/homo-sapiens/c11orf1.html)	1 View

Interactor	Evidence
CERCAM (https://thebiogrid.org/119332/summary/homo-sapiens/cercam.html)	1 View
CH25H (https://thebiogrid.org/114490/summary/homo-sapiens/ch25h.html)	1 View
CHRD (https://thebiogrid.org/114198/summary/homo-sapiens/chrd.html)	1 View
CHRDL2 (https://thebiogrid.org/117395/summary/homo-sapiens/chrdl2.html)	1 View
CIC (https://thebiogrid.org/116767/summary/homo-sapiens/cic.html)	1 View
COL8A1 (https://thebiogrid.org/107692/summary/homo-sapiens/col8a1.html)	1 View
CPSF3L (https://thebiogrid.org/120310/summary/homo-sapiens/cpsf3l.html)	1 View
CTAG2 (https://thebiogrid.org/119058/summary/homo-sapiens/ctag2.html)	1 View
CYSRT1 (https://thebiogrid.org/132001/summary/homo-sapiens/cysrt1.html)	1 View
DPY30 (https://thebiogrid.org/124181/summary/homo-sapiens/dpy30.html)	1 View
EFEMP2 (https://thebiogrid.org/119026/summary/homo-sapiens/efemp2.html)	1 View
ETFDH (https://thebiogrid.org/108411/summary/homo-sapiens/etfdh.html)	1 View
EWSR1 (https://thebiogrid.org/108431/summary/homo-sapiens/ewsr1.html)	1 View
FAM168B (https://thebiogrid.org/126221/summary/homo-sapiens/fam168b.html)	1 View
FAM72B (https://thebiogrid.org/576100/summary/homo-sapiens/fam72b.html)	1 View
FBLN2 (https://thebiogrid.org/108493/summary/homo-sapiens/fbln2.html)	1 View
FBLN5 (https://thebiogrid.org/115771/summary/homo-sapiens/fbln5.html)	1 View
FHL5 (https://thebiogrid.org/114846/summary/homo-sapiens/fhl5.html)	1 View

Interactor	Evidence
GEMIN8P4 (https://thebiogrid.org/138893/summary/homo-sapiens/gemin8p4.html)	1 View
GPRIN2 (https://thebiogrid.org/115070/summary/homo-sapiens/gprin2.html)	1 View
HIST1H4A (https://thebiogrid.org/113955/summary/homo-sapiens/hist1h4a.html)	1 View
HIVEP1 (https://thebiogrid.org/109343/summary/homo-sapiens/hivep1.html)	1 View
KCNK16 (https://thebiogrid.org/123763/summary/homo-sapiens/kcnk16.html)	1 View
KLHL26 (https://thebiogrid.org/120582/summary/homo-sapiens/klhl26.html)	1 View
KPRP (https://thebiogrid.org/138665/summary/homo-sapiens/kprp.html)	1 View
KRT31 (https://thebiogrid.org/110079/summary/homo-sapiens/krt31.html)	1 View
KRT35 (https://thebiogrid.org/110084/summary/homo-sapiens/krt35.html)	1 View
KRT37 (https://thebiogrid.org/114235/summary/homo-sapiens/krt37.html)	1 View
KRT85 (https://thebiogrid.org/110089/summary/homo-sapiens/krt85.html)	1 View
KRTAP1-1 (https://thebiogrid.org/123602/summary/homo-sapiens/krtap1-1.html)	1 View
KRTAP10-3 (https://thebiogrid.org/132133/summary/homo-sapiens/krtap10-3.html)	1 View
KRTAP10-7 (https://thebiogrid.org/132126/summary/homo-sapiens/krtap10-7.html)	1 View
KRTAP11-1 (https://thebiogrid.org/130626/summary/homo-sapiens/krtap11-1.html)	1 View
KRTAP12-1 (https://thebiogrid.org/131687/summary/homo-sapiens/krtap12-1.html)	1 View
KRTAP12-3 (https://thebiogrid.org/132134/summary/homo-sapiens/krtap12-3.html)	1 View
KRTAP13-1 (https://thebiogrid.org/126601/summary/homo-sapiens/krtap13-1.html)	1 View

Interactor	Evidence
KRTAP13-2 (https://thebiogrid.org/130644/summary/homo-sapiens/krtap13-2.html)	1 View
KRTAP13-3 (https://thebiogrid.org/130645/summary/homo-sapiens/krtap13-3.html)	1 View
KRTAP17-1 (https://thebiogrid.org/123813/summary/homo-sapiens/krtap17-1.html)	1 View
KRTAP19-2 (https://thebiogrid.org/130652/summary/homo-sapiens/krtap19-2.html)	1 View
KRTAP19-7 (https://thebiogrid.org/130657/summary/homo-sapiens/krtap19-7.html)	1 View
KRTAP2-3 (https://thebiogrid.org/611123/summary/homo-sapiens/krtap2-3.html)	1 View
KRTAP2-4 (https://thebiogrid.org/124458/summary/homo-sapiens/krtap2-4.html)	1 View
KRTAP22-1 (https://thebiogrid.org/130662/summary/homo-sapiens/krtap22-1.html)	1 View
KRTAP26-1 (https://thebiogrid.org/132867/summary/homo-sapiens/krtap26-1.html)	1 View
KRTAP3-1 (https://thebiogrid.org/123807/summary/homo-sapiens/krtap3-1.html)	1 View
KRTAP4-1 (https://thebiogrid.org/124449/summary/homo-sapiens/krtap4-1.html)	1 View
KRTAP4-2 (https://thebiogrid.org/124455/summary/homo-sapiens/krtap4-2.html)	1 View
KRTAP4-4 (https://thebiogrid.org/124147/summary/homo-sapiens/krtap4-4.html)	1 View
KRTAP4-5 (https://thebiogrid.org/124453/summary/homo-sapiens/krtap4-5.html)	1 View
KRTAP4-7 (https://thebiogrid.org/124451/summary/homo-sapiens/krtap4-7.html)	1 View
KRTAP5-11 (https://thebiogrid.org/136248/summary/homo-sapiens/krtap5-11.html)	1 View
KRTAP5-3 (https://thebiogrid.org/132267/summary/homo-sapiens/krtap5-3.html)	1 View
KRTAP6-1 (https://thebiogrid.org/130649/summary/homo-sapiens/krtap6-1.html)	1 View

Interactor	Evidence
KRTAP6-2 (https://thebiogrid.org/130650/summary/homo-sapiens/krtap6-2.html)	1 View
KRTAP6-3 (https://thebiogrid.org/130651/summary/homo-sapiens/krtap6-3.html)	1 View
KRTAP7-1 (https://thebiogrid.org/130624/summary/homo-sapiens/krtap7-1.html)	1 View
KRTAP9-3 (https://thebiogrid.org/123811/summary/homo-sapiens/krtap9-3.html)	1 View
KRTAP9-4 (https://thebiogrid.org/124444/summary/homo-sapiens/krtap9-4.html)	1 View
KRTAP9-8 (https://thebiogrid.org/123812/summary/homo-sapiens/krtap9-8.html)	1 View
LASP1 (https://thebiogrid.org/110120/summary/homo-sapiens/lasp1.html)	1 View
LCE1C (https://thebiogrid.org/131640/summary/homo-sapiens/lce1c.html)	1 View
LCE1F (https://thebiogrid.org/131643/summary/homo-sapiens/lce1f.html)	1 View
LCE2A (https://thebiogrid.org/131645/summary/homo-sapiens/lce2a.html)	1 View
LCE2B (https://thebiogrid.org/117630/summary/homo-sapiens/lce2b.html)	1 View
LCE2C (https://thebiogrid.org/131646/summary/homo-sapiens/lce2c.html)	1 View
LCE2D (https://thebiogrid.org/131647/summary/homo-sapiens/lce2d.html)	1 View
LCE4A (https://thebiogrid.org/128277/summary/homo-sapiens/lce4a.html)	1 View
LGALS13 (https://thebiogrid.org/118889/summary/homo-sapiens/lgals13.html)	1 View
M1AP (https://thebiogrid.org/126265/summary/homo-sapiens/m1ap.html)	1 View
MGAT5B (https://thebiogrid.org/127000/summary/homo-sapiens/mgat5b.html)	1 View
MIIP (https://thebiogrid.org/121947/summary/homo-sapiens/miip.html)	1 View

Interactor	Evidence
MTNR1B (https://thebiogrid.org/110640/summary/homo-sapiens/mtnr1b.html)	1 View
NBPF19 (https://thebiogrid.org/3190771/summary/homo-sapiens/nbpf19.html)	1 View
NDUFAF6 (https://thebiogrid.org/126481/summary/homo-sapiens/ndufaf6.html)	1 View
NHLRC4 (https://thebiogrid.org/129711/summary/homo-sapiens/nhlrc4.html)	1 View
NOTCH2NL (https://thebiogrid.org/132802/summary/homo-sapiens/notch2nl.html)	1 View
OXER1 (https://thebiogrid.org/127909/summary/homo-sapiens/oxer1.html)	1 View
PCSK5 (https://thebiogrid.org/111152/summary/homo-sapiens/pcsk5.html)	1 View
PLA2G10 (https://thebiogrid.org/113987/summary/homo-sapiens/pla2g10.html)	1 View
PLEKHG4 (https://thebiogrid.org/117402/summary/homo-sapiens/plekhg4.html)	1 View
PLSCR2 (https://thebiogrid.org/121340/summary/homo-sapiens/plscr2.html)	1 View
POU2AF1 (https://thebiogrid.org/111446/summary/homo-sapiens/pou2af1.html)	1 View
PRICKLE4 (https://thebiogrid.org/118997/summary/homo-sapiens/prickle4.html)	1 View
PRR13 (https://thebiogrid.org/119965/summary/homo-sapiens/prr13.html)	1 View
PRR22 (https://thebiogrid.org/127854/summary/homo-sapiens/prr22.html)	1 View
PVRL3 (https://thebiogrid.org/117441/summary/homo-sapiens/pvrl3.html)	1 View
RGS19 (https://thebiogrid.org/115576/summary/homo-sapiens/rgs19.html)	1 View
RNF144B (https://thebiogrid.org/129106/summary/homo-sapiens/rnf144b.html)	1 View
RNF4 (https://thebiogrid.org/111974/summary/homo-sapiens/rnf4.html)	1 View

Interactor	Evidence
RTN4R (https://thebiogrid.org/122388/summary/homo-sapiens/rtn4r.html)	1 View
SLC15A2 (https://thebiogrid.org/112453/summary/homo-sapiens/slc15a2.html)	1 View
SMARCC1 (https://thebiogrid.org/112483/summary/homo-sapiens/smarcc1.html)	1 View
SPRY1 (https://thebiogrid.org/115546/summary/homo-sapiens/spry1.html)	1 View
SPRY4 (https://thebiogrid.org/123599/summary/homo-sapiens/spry4.html)	1 View
SPRYD7 (https://thebiogrid.org/121451/summary/homo-sapiens/spryd7.html)	1 View
TEX37 (https://thebiogrid.org/128332/summary/homo-sapiens/tex37.html)	1 View
TRIM31 (https://thebiogrid.org/116257/summary/homo-sapiens/trim31.html)	1 View
TRIM42 (https://thebiogrid.org/130425/summary/homo-sapiens/trim42.html)	1 View
TRIP6 (https://thebiogrid.org/113056/summary/homo-sapiens/trip6.html)	1 View
TSPAN4 (https://thebiogrid.org/112961/summary/homo-sapiens/tspan4.html)	1 View
UBAP2 (https://thebiogrid.org/120938/summary/homo-sapiens/ubap2.html)	1 View
USP54 (https://thebiogrid.org/127739/summary/homo-sapiens/usp54.html)	1 View
VWC2 (https://thebiogrid.org/131984/summary/homo-sapiens/vwc2.html)	1 View
WISP1 (https://thebiogrid.org/114367/summary/homo-sapiens/wisp1.html)	1 View
WWOX (https://thebiogrid.org/119707/summary/homo-sapiens/wwox.html)	1 View
YPEL3 (https://thebiogrid.org/123740/summary/homo-sapiens/ypel3.html)	1 View
ZBTB42 (https://thebiogrid.org/612563/summary/homo-sapiens/zbtb42.html)	1 View

Interactor	Evidence
ZNF330 (https://thebiogrid.org/118131/summary/homo-sapiens/znf330.html)	1 View
ZNF34 (https://thebiogrid.org/123310/summary/homo-sapiens/znf34.html)	1 View
ZRANB1 (https://thebiogrid.org/120139/summary/homo-sapiens/zranb1.html)	1 View

Previous 1 Next

Copyright © 2023 TyersLab.com (http://www.tyerslab.com), All Rights Reserved.

Terms and Conditions (https://wiki.thebiogrid.org/doku.php/terms_and_conditions) | Privacy Policy (https://wiki.thebiogrid.org/doku.php/privacy_policy) | CRISPR Database (https://orcs.thebiogrid.org) | Osprey (https://osprey.thebiogrid.org) | Yeast Kinome (https://thebiogrid.org/project/2) | TyersLab.com (http://www.tyerslab.com) | SGD (https://www.yeastgenome.org) | GitHub (https://github.com/BioGRID) | YouTube (https://www.youtube.com /user/TheBioGRID) | Twitter (https://twitter.com/biogrid)
