# Supplementary material for: OTX Genes in Adult Tissues
Source: Int J Mol Sci. 2023 Nov 30;24(23):16962. doi: 10.3390/ijms242316962 (PMC10707059; doi:10.3390/ijms242316962)
Supplement: Supplementary file 1 [file ijms-24-16962-s001.zip › Supplementary Figure 2 - OTX2 Result Summary BioGRID.pdf]

BioGRID COVID-19 Coronavirus Curation Project (https://thebiogrid.org/project/3)
Search BioGRID for SARS-CoV-2 Protein Interactions (https://thebiogrid.org/search.php?search=SARS-CoV-2*&organism=2697049) | Download SARS-CoV-2 and Coronavirus-Related Interactions (https://downloads.thebiogrid.org/File/BioGRID/Latest-Release/BIOGRID-PROJECT-covid19_coronavirus_project-LATEST.zip)

OTX2

Homo sapiens

CPHD6, MCOPS5

orthodenticle homeobox 2

- CRISPR Database (https://orcs.thebiogrid.org/Gene/5015)
- OMIM (http://www.ncbi.nlm.nih.gov/omim/600037)
- HGNC (https://www.genenames.org/data/gene-symbol-report/#!/hgnc_id/HGNC:8522)
- Alliance of Genome Resources (https://www.alliancegenome.org/gene/HGNC:8522)
- Entrez Gene (http://www.ncbi.nlm.nih.gov/gene/5015)
- RefSeq (http://www.ncbi.nlm.nih.gov/sites/entrez?db=protein&cmd=DetailsSearch&term=NP_068374+OR+NP_758840+OR+NP_001257452+OR+NP_001257453+OR+NP_001257454)
- UniprotKB (http://www.uniprot.org/uniprot/P32243)
- HPRD (http://www.hprd.org/protein/07190)

Download Curated Data for this Protein

Switch View: Interactors 67 Interactions 70 Network

Showing **1** to **67** of **67** unique interactors

Filter Interactions... ADV

Interactor	Evidence
APP (https://thebiogrid.org/106848/summary/homo-sapiens/app.html)	2 View
ATXN1 (https://thebiogrid.org/112217/summary/homo-sapiens/atxn1.html)	2 View
FOXA2 (https://thebiogrid.org/109412/summary/homo-sapiens/foxa2.html)	2 View

Interactor	Evidence
ASIC4 (https://thebiogrid.org/120693/summary/homo-sapiens/asic4.html)	1 View
ATP6V1G3 (https://thebiogrid.org/126042/summary/homo-sapiens/atp6v1g3.html)	1 View
AURKC (https://thebiogrid.org/112671/summary/homo-sapiens/aurkc.html)	1 View
B4GALNT2 (https://thebiogrid.org/125896/summary/homo-sapiens/b4galnt2.html)	1 View
BCOR (https://thebiogrid.org/120228/summary/homo-sapiens/bcor.html)	1 View
BNIP2 (https://thebiogrid.org/107131/summary/homo-sapiens/bnip2.html)	1 View
C11ORF73 (https://thebiogrid.org/119574/summary/homo-sapiens/c11orf73.html)	1 View
CDK3 (https://thebiogrid.org/107453/summary/homo-sapiens/cdk3.html)	1 View
CDK4 (https://thebiogrid.org/107454/summary/homo-sapiens/cdk4.html)	1 View
CREB1 (https://thebiogrid.org/107775/summary/homo-sapiens/creb1.html)	1 View
DDX46 (https://thebiogrid.org/115210/summary/homo-sapiens/ddx46.html)	1 View
DDX49 (https://thebiogrid.org/120040/summary/homo-sapiens/ddx49.html)	1 View
DHX16 (https://thebiogrid.org/114027/summary/homo-sapiens/dhx16.html)	1 View
DHX36 (https://thebiogrid.org/128022/summary/homo-sapiens/dhx36.html)	1 View
EIF5B (https://thebiogrid.org/115024/summary/homo-sapiens/eif5b.html)	1 View
FAM46B (https://thebiogrid.org/125440/summary/homo-sapiens/fam46b.html)	1 View
GPR45 (https://thebiogrid.org/116411/summary/homo-sapiens/gpr45.html)	1 View
GTF2F2 (https://thebiogrid.org/109218/summary/homo-sapiens/gtf2f2.html)	1 View

Interactor	Evidence
GUCA1B (https://thebiogrid.org/109234/summary/homo-sapiens/guca1b.html)	1 View
H2AFX (https://thebiogrid.org/109268/summary/homo-sapiens/h2afx.html)	1 View
HN1 (https://thebiogrid.org/119338/summary/homo-sapiens/hn1.html)	1 View
HNF1B (https://thebiogrid.org/112790/summary/homo-sapiens/hnf1b.html)	1 View
HOMER3 (https://thebiogrid.org/114843/summary/homo-sapiens/homer3.html)	1 View
HSD3B7 (https://thebiogrid.org/123208/summary/homo-sapiens/hsd3b7.html)	1 View
HSPA8 (https://thebiogrid.org/109544/summary/homo-sapiens/hspa8.html)	1 View
IQGAP1 (https://thebiogrid.org/114353/summary/homo-sapiens/iqgap1.html)	1 View
IWS1 (https://thebiogrid.org/120807/summary/homo-sapiens/iws1.html)	1 View
KIF2A (https://thebiogrid.org/109997/summary/homo-sapiens/kif2a.html)	1 View
KLF3 (https://thebiogrid.org/119426/summary/homo-sapiens/klf3.html)	1 View
LHX1 (https://thebiogrid.org/110163/summary/homo-sapiens/lhx1.html)	1 View
M1AP (https://thebiogrid.org/126265/summary/homo-sapiens/m1ap.html)	1 View
MAP7 (https://thebiogrid.org/114515/summary/homo-sapiens/map7.html)	1 View
NAA50 (https://thebiogrid.org/123185/summary/homo-sapiens/naa50.html)	1 View
NANOG (https://thebiogrid.org/123000/summary/homo-sapiens/nanog.html)	1 View
NELFCD (https://thebiogrid.org/119572/summary/homo-sapiens/nelfcd.html)	1 View
NFYC (https://thebiogrid.org/110868/summary/homo-sapiens/nfyc.html)	1 View

Interactor	Evidence
NSP16 (https://thebiogrid.org/4383864/summary/severe-acute-respiratory-syndrome-coronavirus-2/nsp16.html)	1 View
OR1L3 (https://thebiogrid.org/117803/summary/homo-sapiens/or1l3.html)	1 View
OR6C1 (https://thebiogrid.org/133512/summary/homo-sapiens/or6c1.html)	1 View
OTX2 (https://thebiogrid.org/111055/summary/homo-sapiens/otx2.html)	1 View
PDK1 (https://thebiogrid.org/111189/summary/homo-sapiens/pdk1.html)	1 View
PINX1 (https://thebiogrid.org/120319/summary/homo-sapiens/pinx1.html)	1 View
POLDIP3 (https://thebiogrid.org/123998/summary/homo-sapiens/poldip3.html)	1 View
POU5F1 (https://thebiogrid.org/111456/summary/homo-sapiens/pou5f1.html)	1 View
RBM47 (https://thebiogrid.org/119998/summary/homo-sapiens/rbm47.html)	1 View
RBMXL1 (https://thebiogrid.org/138936/summary/homo-sapiens/rbmxl1.html)	1 View
RNASE11 (https://thebiogrid.org/125785/summary/homo-sapiens/rnase11.html)	1 View
RPS26 (https://thebiogrid.org/112145/summary/homo-sapiens/rps26.html)	1 View
RTCA (https://thebiogrid.org/114187/summary/homo-sapiens/rtca.html)	1 View
SMARCB1 (https://thebiogrid.org/112482/summary/homo-sapiens/smarcb1.html)	1 View
SNRPA (https://thebiogrid.org/112510/summary/homo-sapiens/snrpa.html)	1 View
SUGP1 (https://thebiogrid.org/121767/summary/homo-sapiens/sugp1.html)	1 View
TBCEL (https://thebiogrid.org/128592/summary/homo-sapiens/tbcel.html)	1 View
TMEM11 (https://thebiogrid.org/114361/summary/homo-sapiens/tmem11.html)	1 View

Interactor	Evidence
TMPO (https://thebiogrid.org/112967/summary/homo-sapiens/tmpo.html)	1 View
TNPO2 (https://thebiogrid.org/119024/summary/homo-sapiens/tnpo2.html)	1 View
TRMT10B (https://thebiogrid.org/127659/summary/homo-sapiens/trmt10b.html)	1 View
WRN (https://thebiogrid.org/113323/summary/homo-sapiens/wrn.html)	1 View
ZC3H10 (https://thebiogrid.org/124317/summary/homo-sapiens/zc3h10.html)	1 View
ZDHHC17 (https://thebiogrid.org/116965/summary/homo-sapiens/zdhhc17.html)	1 View
ZMAT2 (https://thebiogrid.org/127499/summary/homo-sapiens/zmat2.html)	1 View
ZNF765 (https://thebiogrid.org/124860/summary/homo-sapiens/znf765.html)	1 View
ZNHIT1 (https://thebiogrid.org/115730/summary/homo-sapiens/znhit1.html)	1 View
SREBF2 (https://thebiogrid.org/112599/summary/homo-sapiens/srebf2.html)	1 View

Previous 1 Next

Copyright © 2023 TyersLab.com (http://www.tyerslab.com), All Rights Reserved.

Terms and Conditions (https://wiki.thebiogrid.org/doku.php/terms_and_conditions) | Privacy Policy (https://wiki.thebiogrid.org/doku.php/privacy_policy) | CRISPR Database (https://orcs.thebiogrid.org) | Osprey (https://osprey.thebiogrid.org) | Yeast Kinome (https://thebiogrid.org/project/2) | TyersLab.com (http://www.tyerslab.com) | SGD (https://www.yeastgenome.org) | GitHub (https://github.com/BioGRID) | YouTube (https://www.youtube.com /user/TheBioGRID) | Twitter (https://twitter.com/biogrid)
